# Effects of Danshensu on Platelet Aggregation and Thrombosis: *In Vivo* Arteriovenous Shunt and Venous Thrombosis Models in Rats

**DOI:** 10.1371/journal.pone.0110124

**Published:** 2014-11-06

**Authors:** Chen Yu, Dong Qi, Wei Lian, Qing-Zhong Li, Hong-Juan Li, Hua-Ying Fan

**Affiliations:** 1 School of Pharmacy, Binzhou Medical University, Yantai, Shandong, China; 2 Department of Nephrology, Yantai Yu-Huang-Ding/Qingdao University Hospital, Yantai, Shandong, China; 3 Yantai Yan-Tai-Shan Hospital, Yantai, Shandong, China; 4 School of Pharmacy, Yantai University, Yantai, Shandong, China; IIBB-CSIC-IDIBAPS, Spain

## Abstract

Danshensu, a type of dihydroxyphenyl lactic acid, is one of the most abundant active phenolic acids in the dried root of *Salvia miltiorrhizae* (Lamiaceae)—widely used traditional Chinese medicine. The effects of danshensu on platelet aggregation and thrombus formation in rats were examined using various methods. It was found that danshensu significantly reduced thrombus weight in 2 experimental thrombosis models; dose-dependent inhibition of adenosine diphosphate (ADP) and arachidonic acid (AA)-induced platelet aggregation occurred in normal and blood stasis-induced rats; Danshensu also significantly mitigated blood viscosity, plasma viscosity and hematocrit levels. Moreover, danshensu significantly inhibited venous thrombosis-induced expression of cyclooxygenases-2 (COX-2) rather than cyclooxygenases-1(COX-1) in the venous walls, down regulated thromboxane B_2_ (TXB_2_) and up regulated 6-keto prostaglandin F_1α_ (6-keto-PGF_1α_), normalizing the TXB_2_/6-keto-PGF_1α_ ratio. In addition, danshensu did not induce gastric lesions and even had protective effects on aspirin-induced ulcer formation at doses as high as 60 mg/kg. These findings suggest that the antithrombotic and antiplatelet aggregation effects of danshensu are attributed to its highly selective inhibition of COX-2 and ability to normalize the thromboxane A_2_(TXA_2_)/prostacyclin(PGI_2_) balance. These findings suggest that danshensu have great prospects in antithrombotic and antiplatelet therapy.

## Introduction

Thrombus formation accelerates the progression of various cardiovascular and cerebrovascular disorders. Thromboembolic complications of arteriosclerosis, heart attack, stroke and peripheral vascular disease are the principal causes of death in developed countries. Arterial and venous thrombi comprises primarily platelet aggregates.

Platelets mediate the initiation of thrombosis through platelet adhesion, activation and aggregation [1]. A collaborative meta-analysis of randomized trials has demonstrated that antiplatelet therapy is beneficial in treating thromboembolic diseases and preventing serious vascular events, arterial occlusion and venous thromboembolism in a wide range of patients who are at high risk for occlusive vascular events [2]. Thus, the inhibition of platelet function has the potential to treat circulatory diseases, spurring the development of many antiplatelet and antithrombotic agents and prompting an examination of their effects in preventing thrombosis.

Danshen, the dried root of *Salvia miltiorrhizae* (Lamiaceae), is one of the most widely used traditional herbal medicines against various cardiovascular and cerebrovascular diseases, including coronary heart disease, myocardial infarction, stroke and hypertension. Danshen contains hydrophilic phenolics and lipophilic quinones, which have many pharmacological and therapeutic properties [3].

**Figure 1:**
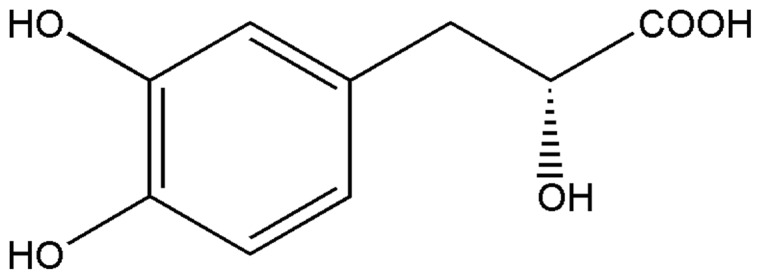
Chemical structure of danshensu (C_9_H_10_O_5_, molecular weight  = 198.17).

Increasing data have demonstrated that danshensu is an abundant and representative phenolic acid in danshen [4]. Danshensu (chemical structure shown in Fig. 1)—known as β-3,4-dihydroxyphenyl-lactic acid—has several mechanisms by which it elicits its therapeutic effects. Danshensu can mitigate cardiovascular and cerebrovascular disturbances [5] by improving microcirculation, increasing blood flow, relaxing the coronary arteries and promoting anticoagulation [6].

However, the antithrombotic and antiplatelet effects and mechanisms of danshensu are unknown. The purpose of this study was to determine the effects and mechanisms of danshensu with regard to platelet aggregation, thrombus formation and blood circulation so as to provide pharmacological evidence that supports its clinical application.

## Results

### Effects on platelet aggregation *in vivo*

**Figure 2:**
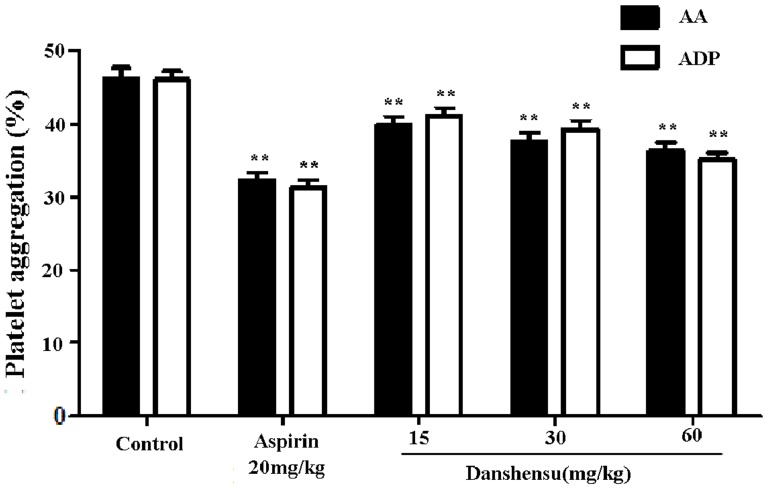
Effect of danshensu on platelet aggregation in normal rats. Blood was drawn 60 min after intragastric danshensu administration. Platelet aggregation was induced by diphosphate (ADP) and arachidonic acid (AA). Data are expressed as mean ± SEM (each group, n = 10). **P<0.01 with control group.

To assess the effect of danshensu on platelet aggregation, we measured the inhibition of platelet aggregation on danshensu induced by ADP and AA in normal rats.As shown in Fig. 2, compared with the control group, ADP and AA-induced platelet aggregation was altered by pretreatment with danshensu and aspirin. Danshensu dose-dependently inhibited AA-induced platelet aggregation by approximately 13.9% at 15 mg/kg, 17.9% at 30 mg/kg and 21.5% at 60 mg/kg, respectively. This result showed that similar inhibition in the ADP-induced platelet aggregation.The inhibition of platelet aggregation induced by ADP was 10.5%, 14.6% and 23.5% at doses of 15, 30, and 60 mg/kg, respectively.

**Figure 3:**
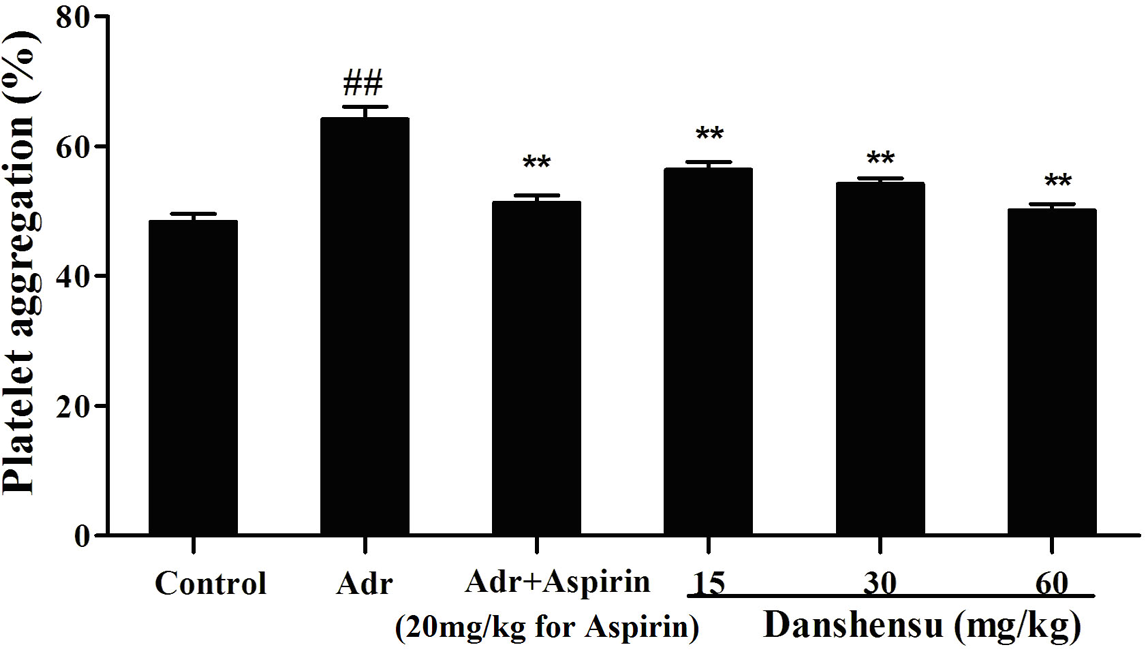
Effect of danshensu on platelet aggregation in blood stasis rats. Blood was drawn 60 min after intragastric danshensu administration. Platelet aggregation was induced by arachidonic acid (AA). Data are expressed as mean ± SEM (each group, n = 10). The blood stasis model was built during the interval between when 2 injections of adrenaline hydrochloride (Adr) were given to rats placed in ice-cold water. *##P*<0.01 compared with normal control. *P<0.05 **P<0.01 compared with Adr control.

Further, we determined effect of danshensu on platelet aggregation in blood stasis rats induced by AA. Blood stasis increased platelet aggregation compared with the control group as shown in Fig. 3, which was also modified by pretreatment with danshensu in a dose-dependent manner. This inhibitory effect of danshensu at dose of 60 mg/kg was comparable with that of aspirin at 20 mg/kg in blood stasis rats.

### Effect on thrombus formation

**Figure 4:**
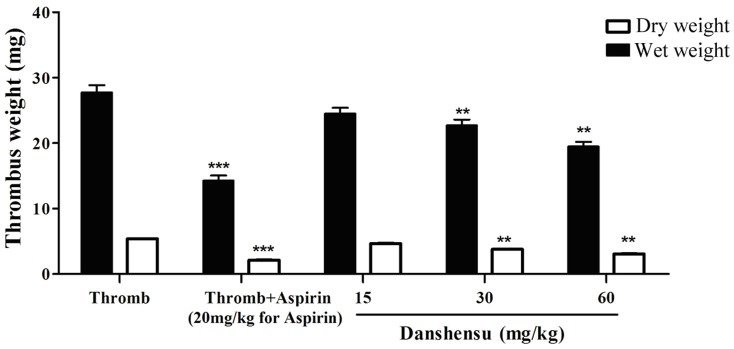
Antithrombotic activity of danshensu in the rat model of arteriovenous shunt. The drug or solvent was administered orally 60 min before thrombogenic challenge. Data are expressed as mean ± SEM (each group, n = 10). Thromb: thrombosis. *P<0.05, **P<0.01, ***P<0.001 compared with thrombotic control group.

Two rat models of thrombosis were developed by installing an arteriovenous shunt and ligating the inferior vena cava to study the antithrombotic activity and mechanism of danshensu *in vivo*. Pretreatment with danshensu (15 to 60 mg/kg) decreased thrombus formation dose-dependently in the arteriovenous shunt model.The inhibition of thrombus formation was about 12.6%, 18.2% and 29.6% respectively (Fig. 4).

**Figure 5:**
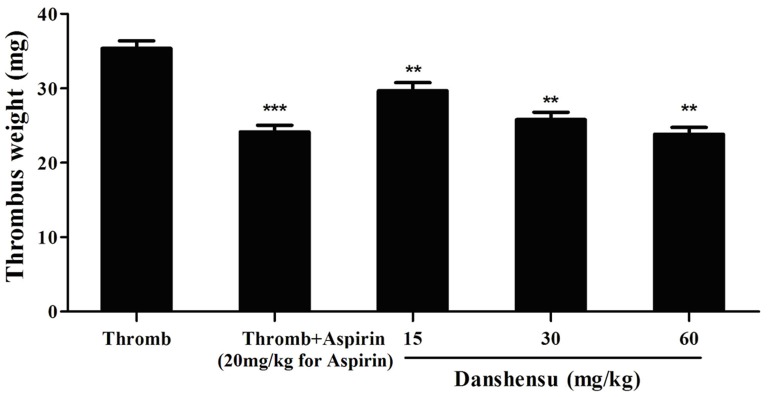
Antithrombotic activity of danshensu in the rat model of ligating inferior vena cava. The drug or solvent was administered orally 60 min before the thrombogenic challenge. Data are expressed as mean ± SEM (each group, n = 10). Thromb: thrombosis. *P<0.05, **P<0.01, ***P<0.001 compared with thrombotic control group.

As shown in Fig. 5, this result was confirmed by inferior vena cava model. Residual thrombi from the occlusive vessels of all rats were measured. Compared with the thrombotic control group, aspirin at 20 mg/kg decreased the thrombus weight from 35.37±3.03 mg to 24.14±4.23 mg versus 29.65±5.62 mg with 15 mg/kg, 25.78±3.95 mg with 30 mg/kg and 23.84±2.26 mg with 60 mg/kg of danshensu, respectively. Antithrombotic activity of danshensu at dose of 60 mg/kg is comparable with that of aspirin at 20 mg/kg.

### Effects on COX and possible mechanisms

**Figure 6:**
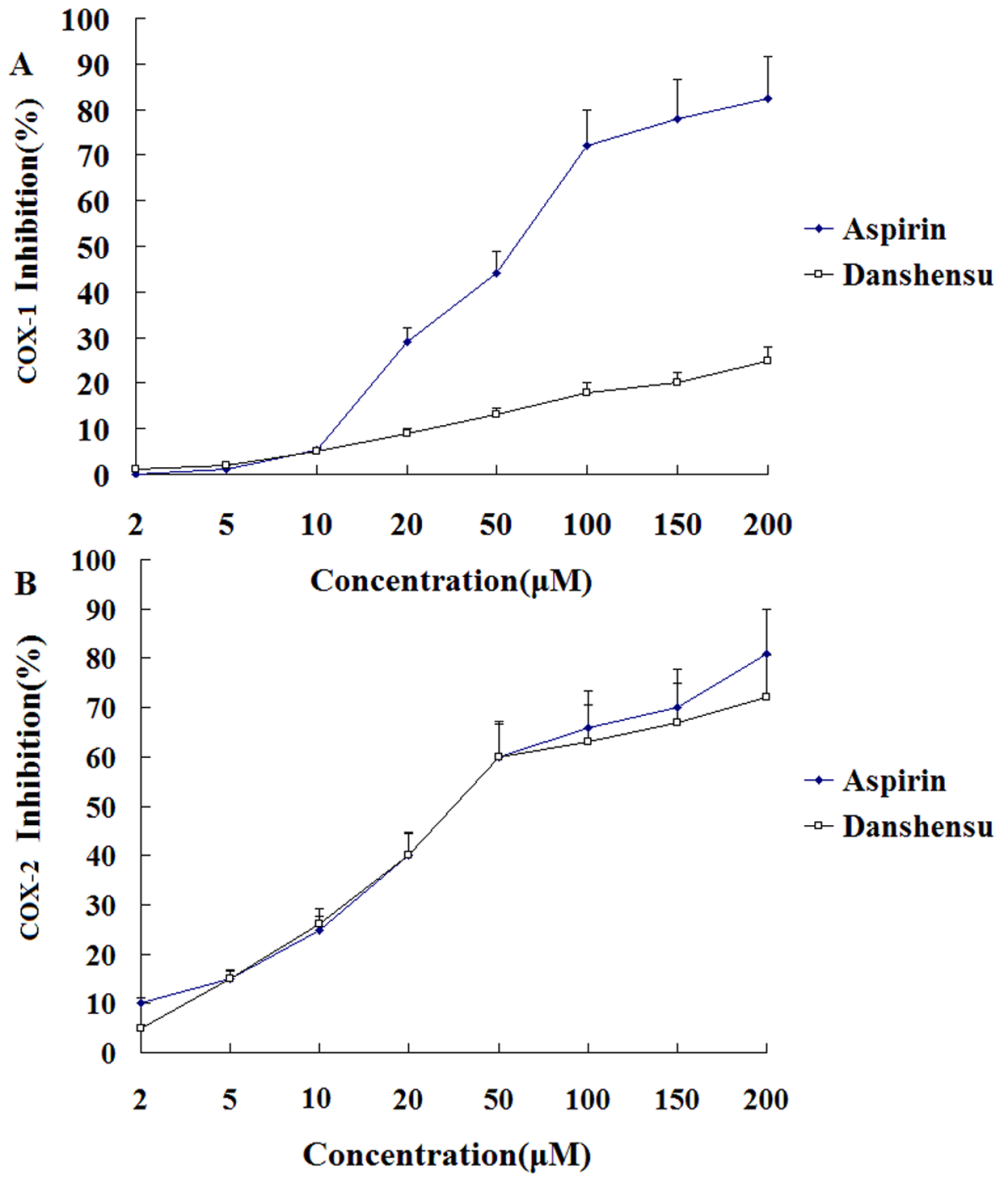
Direct effect of various concentrations of danshensu and aspirin on the enzymatic activities of COX-1 and COX-2. Danshensu and aspirin (water, as a control) were incubated with COX-1 (A) or COX-2 (B) for 10 min and then AA was added. COX enzyme activities are reflected by the amount of PGE_2_ produced. PGE_2_ production was measured by ELISA. Enzyme activity in the sample without treatment (water alone) served as control and results are expressed as inhibitory rate of COX-1 and COX-2 activity compared with solvent control.

Inhibitory effects of danshensu and aspirin against 2 cyclooxygenases (COX) isoforms were first examined *in vitro* using enzymatic assays. While danshensu had little inhibitory effect on COX-1, danshensu decreased COX-2 activity at 20 to 200 µM even at 200 µM yielding an IC_50_ value of approximately 44 µM (Fig. 6 A vs B).

In contrast, aspirin inhibited COX-1 and COX-2, with geometric mean IC_50_ values of 39.8 and 60.7 µM, respectively. The IC_50_ ratios of COX-1 to COX-2 for danshensu and aspirin exceeded 10 and 0.67 respectively, it is demonstrated that danshensu selectively inhibits COX-2 over COX-1 by approximately 20-fold.

**Figure 7:**
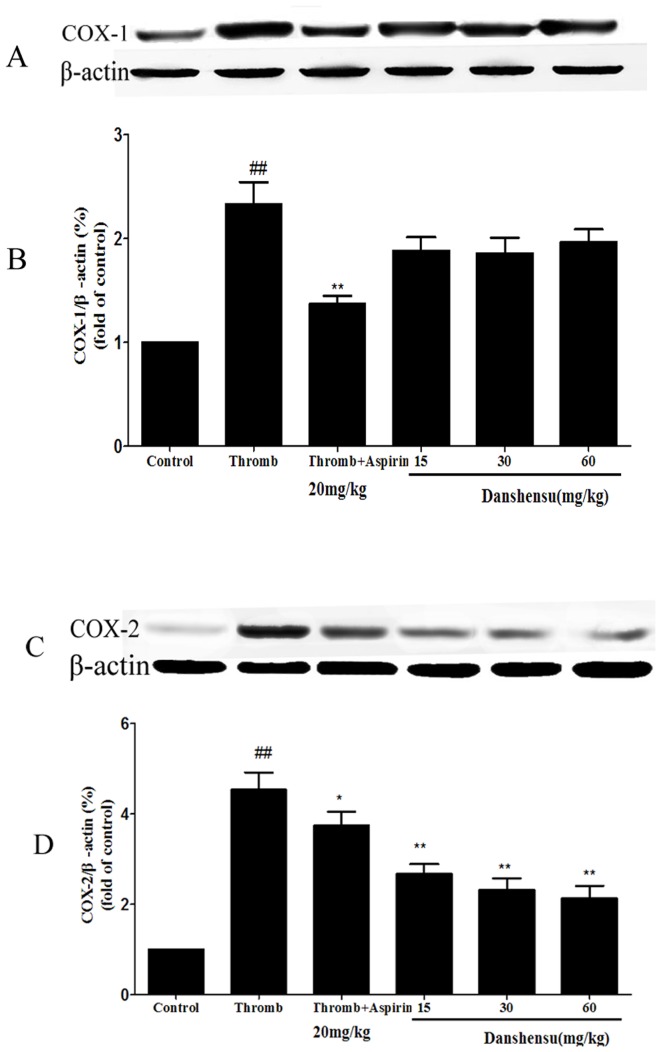
The effects of danshensu on COX expression. Rats were given 15, 30 and 60 mg/kg danshensu intragastrically and 20 mg/kg aspirin for 7 days. Venous thrombus formation was induced by inferior vena cava (IVC) ligation to produce thrombus after last administration.Vein walls were harvested from venous thrombosis and the expression of COX-1 and COX-2 was assessed by western analysis (A and C). β-actin was measured to confirm equal loading of proteins. Densitometric analysis of COX-1 (B) and COX-2 (D) expression is represented by the mean from 3 separate experiments. Data were normalized to β-actin levels. Thromb: thrombosis. *##P*<0.01 compared with normal control. *P<0.05, **P<0.01 compared with thrombotic control group.

The effects of danshensu on COX-1 and COX-2 levels were examined using western blot technique. As shown in Fig. 7, the expression of COX-1 and COX-2 induced by inferior vena cava ligation was significantly elevated when compared with control group. It is clear that aspirin significantly inhibited both COX-1 and COX-2 expression while danshenu only significantly inhibited COX-2 but not COX-1 expression (Fig. 7B vs D). This observation indicates that danshensu exerts its antiplatelet effect by selectively targeting on COX-2.

Next, we measured the serum concentrations of TXB_2_ and 6-keto-PGF1α in the rats. TXB_2_ and 6-keto-PGF_1α_ levels increased significantly, accompanying a rise in the TXB_2_/6-keto-PGF_1α_ ratio in the 2 thrombosis models. Aspirin significantly reduced TXB_2_ and 6-keto-PGF_1α_ levels, decreasing the TXB_2_/6-keto-PGF_1α_ ratio. Danshensu downregulated TXB_2_ and increased 6-keto-PGF_1α_ levels, especially at 30 and 60 mg/kg, normalizing the TXB_2_/6-keto-PGF_1α_ ratio better than aspirin (Tables 1 and 2).

**Table 1:** Effects of danshensu on plasma TXB_2_ and 6-keto-PGF_1α_ levels and TXB_2_-6-keto-PGF_1α_ ratio in rats in the arteriovenous shunt model.

Group	Dose(mg/kg)	TXB_2_ (pg/mL)	6-keto-PGF_1α_ (pg/mL)	TXB_2_/6-keto-PGF_1α_
Normal		180.0±8.3	487.3±16.6	0.38±0.01
Thromb		552.2±18.9^##^	602.5±20.4^##^	0.93±0.03^##^
Thromb+Aspirin	20	56.5±1.9***	190.5±7.5***	0.30±0.01***
Thromb +Danshensu	15	258.1±10.2**	621.7±19.8*	0.40±0.01**
	30	228.7±11.5**	638.3±22.1**	0.37±0.02**
	60	206.4±8.9**	649.2±15.8**	0.35±0.02**

Blood was drawn from the abdominal aorta at the end of arteriovenous shunt test period and anticoagulated with indomethacin-EDTA-Na_2_. Plasma samples were prepared by centrifugation and analyzed by radioimmunoassay. Data are expressed as mean±SEM (each group, n = 10). ^#^ *P*<0.05, ^##^ *P*<0.01 compared with normal group, *P<0.05, **P<0.01, ***P<0.001 compared with thrombotic control group.

**Table 2:** Effects of danshensu on plasma TXB_2_ and 6-keto-PGF_1α_ levels and TXB_2_-6-keto-PGF_1α_ ratio in rats the venous thrombosis model.

Group	Dose(mg/kg)	TXB_2_ (pg/mL)	6-keto-PGF_1α_ (pg/mL)	TXB_2_/6-keto-PGF_1α_
Normal		197.0±8.2	528.3±16.8	0.37±0.03
Thromb		605.2±21.9^##^	652.5±24.6^##^	0.95±0.03^##^
Thromb+Aspirin	20	106.5±4.2***	395.0±14.5***	0.29±0.01***
Thromb +Danshensu	15	358.1±12.8**	663.7±24.2*	0.50±0.02**
	30	328.7±10.6**	684.3±25.1**	0.45±0.02**
	60	306.4±8.2***	719.2±20.9**	0.40±0.02**

Blood was drawn from the abdominal aorta at the end of venous thrombus formation period and anticoagulated with indomethacin-EDTA-Na_2_. Plasma samples were prepared by centrifugation and analzyed by radioimmunoassay. Data are expressed as mean±SEM (each group, n = 10). ^#^ *P*<0.05, ^##^ *P*<0.01 compared with normal group, *P<0.05, **P<0.01, ***P<0.001 compared with thrombotic control group.

### Effect on hemorheology

whole blood viscosity (WBV) at all shear rates, plasma viscosity (PV) and hematocrit values (Hct) significantly rose in blood stasis rats, but danshensu significantly reduced blood viscosity, plasma viscosity and hematocrit, particularly at 30 and 60 mg/kg (Table 3).

**Table 3:** The effect of danshensu on the hemorheological parameters in rats.

Group	WBV(mPa.s)			PV(mPa.s)	Hct(%)
	Low shear rate	Medium shear rate	High shear rate		
Normal Control	22.72±1.21	5.88±0.25	4.44±0.11	1.41±0.08	0.41±0.02
Adr Control	39.55±1.35^##^	9.01±0.32^##^	6.52±0.23^##^	2.06±0.06^##^	0.45±0.02^##^
Adr Aspirin 20 mg/kg	26.59±0.87**	6.82±0.21**	5.02±0.19**	1.66±0.06*	0.44±0.04
Adr Danshensu 15 mg/kg	35.36±2.25*	8.64±0.51*	6.28±0.31*	1.89±0.08*	0.45±0.02*
Adr Danshensu 30 mg/kg	31.24±1.08**	7.74±0.23**	5.65±0.18**	1.67±0.09*	0.44±0.02*
Adr Danshensu 60 mg/kg	27.12±1.05**	6.65±0.19**	4.78±0.12**	1.45±0.42**	0.43±0.02*

Rats were treated by subcutaneous injection of adrenaline hydrochloride and cold stress. The normal control rats received only saline. Next, blood was collected 30 min after drug administration. Hemorheologic parameters were analyzed by routine laboratory assays. Data are expressed as mean±SEM (each group, n = 10). #P<0.05, ##P<0.01 compared with normal control. *P<0.05 **P<0.01 compared with Adr control. Adr: adrenaline hydrochloride, WBV: whole blood viscosity, PV: plasma viscosity, Hct: hematocrit.

### Protective effects of danshensu on gastric mucosa

**Figure 8:**
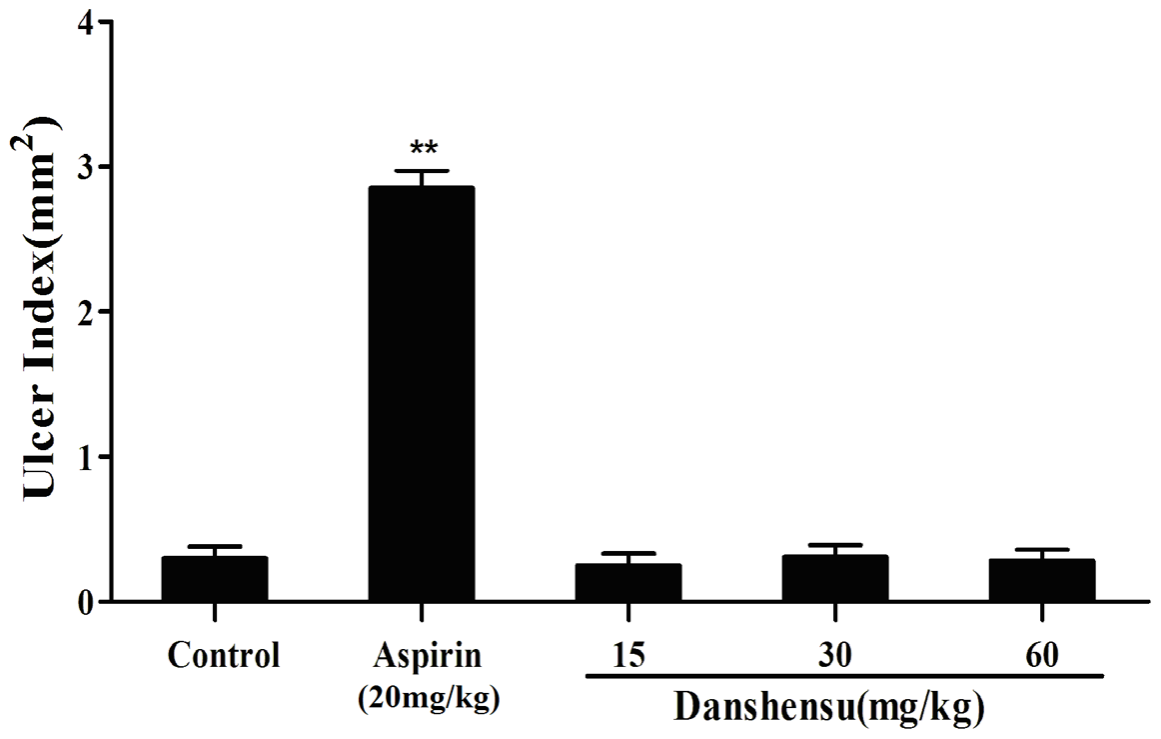
Gastric ulcerogenic response induced by danshensu and aspirin in rats. The test compounds were administered orally at the indicated dose (mg/kg) to rats for 7 days. The animals were sacrificed after the last drug administration and the total length of mucosal lesions in each stomach was used to create an ulcer index. Data are presented as the mean ± SEM (each group, n = 10). **P<0.01 versus the control group.

We examined the gastrointestinal (GI) effects of oral administration of danshensu and aspirin in rats for 7 days. The ulcer index for all groups is summarized in Fig. 8. Aspirin resulted in gastric lesions and the ulcer index was significantly higher than the control group. In contrast, danshensu did not induce gastric lesions, even at as high as 60 mg/kg.

**Figure 9:**
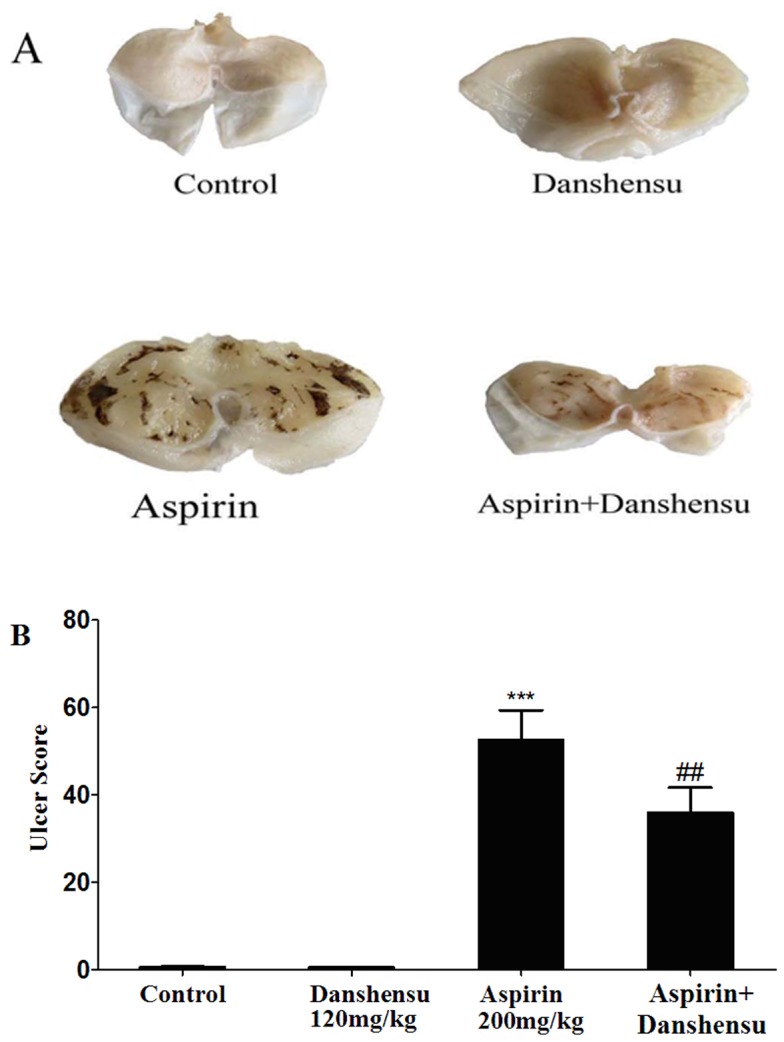
Effects of danshensu on aspirin-induced gastric lesions in mice. A: Photographs of gastric mucosa in the control group and danshensu, aspirin and aspirin + danshensu treatment groups are shown. B: The hemorrhagic ulcer index (mm^2^) for each condition. Values are shown as the mean ± SEM(each group, n = 10). **P<0.01 compared with the control rats. *##P*<0.01 compared with the aspirin-treated rats.

We also investigated the effect of danshensu on aspirin-induced gastric lesions. The macroscopic findings of open stomachs are shown in Fig. 9A, we found that oral administration of aspirin (200 mg/kg body weight) induced severe mucosal damage in the gastric corpus of mice. Danshensu had no damaging effects on the stomach. In addition co-administration of danshensu with aspirin could inhibit aspirin-induced ulcer formation. Fig. 9B shows the ulcer score of gastric hemorrhagic ulcers in each group. These data suggest that danshensu has gastroprotective activities and it also prevents aspirin-induced gastric ulcer formation.

## Discussion

Many life-threatening diseases, such as atherosclerosis, cerebrovascular thrombosis, coronary artery disease, stroke and tumor metastasis are related with platelet dysfunctions [7]. Many available antiplatelet agents interfere with platelet function at various levels of activation, which results in several clinical disadvantages, including gastrointestinal side effects and hemorrhage. For this reason, a search for safer and more effective antiplatelet agents without these adverse effects would be highly desirable. In recent years, new therapeutic agents have been derived from Chinese herbs and there is growing interest in this area.

In this study, we examined the antiplatelet and antithrombotic effects of danshensu and the pharmacological mechanisms. We determined effects of danshensu on platelet function by measuring ADP and AA-induced platelet aggregation. Our results showed that danshensu produced marked antiplatelet effect on ADP and AA platelet agonists ex vivo aggregation. This inhibitory effect of danshensu at dose of 60 mg/kg was comparable with aspirin at 20 mg/kg in blood stasis-induced rats.

The effects of danshensu on thrombus formation were studied in arteriovenous shunt model and inferior vena cava model; these models simulate arterial and venous thrombosis observed in humans [8]. Our results demonstrated that danshensu has potent antithrombotic effects against arterial and venous thrombosis.

To assess the effect of danshensu on microcirculation, we evaluated hemorheologic parameters in blood stasis model. Blood stasis can result in platelet aggregation and hemorheological abnormalities. Hemorheological disorders mediate the pathogenesis and development of many cardiovascular and cerebrovascular diseases [9]. In this study, rats were injected subcutaneously with adrenaline hydrochloride (Adr) and treated with cold stress. As a result, blood viscosity, plasma viscosity and hematocrit increased significantly compared with the control group. Our results suggest that Adr combined with exposure to ice-cold water induces blood stasis resulting in hemorheological abnormalities. Platelet aggregation is believed to be a factor that determines blood viscosity [10], consistent with our results that danshensu improves hemorheologic parameters, ameliorates blood stasis and promotes circulation by decreasing whole blood viscosity, secondary to the inhibitory effect on platelet aggregation.

In our study, Danshensu exhibited apparent antiplatelet and antithrombotic activity. In order to explore the further mechanisms of antiplatelet and anti-thrombosis, the activity and expression of COXs were measured. We also evaluated the levels of TXB_2_ and 6-keto-PGF_1α_ in both arterial and venous thrombosis models. Our results indicated that danshensu could selectively inhibit COX-2 rather than COX-1 to regulate the balance of TXA_2_/PGI_2_. Therefore, danshensu exhibited superior antiplatelet and antithrombotic effects compared with aspirin.

Disruption of the TXA_2_-PGI_2_ balance increases the risk of thrombosis [11]. TXA_2_ and PGI_2_ are metabolites of arachidonic acid, which is hydrolyzed by COXs and transformed into endoperoxides, prostaglandins (PGs) and TXA_2_ [12]. COXs exist as 2 distinct isoforms: COX-1 and COX-2. COX-1 which is constitutively expressed in most tissues and has high levels of activity in the the gastrointestinal tract, is thought to exert homeostatic properties that are crucial for gastric physiologic function, including mucosal protectionwhereas COX-2 is absent from most healthy tissues but is induced by proinflammatory and proliferative stimuli after exposure to cytokines, immunological stimuli and growth factors [13], [14]. TXA_2_ is a potent inducer of platelet aggregation and vasoconstriction and its levels rise in thrombus models. In contrast, PGI_2_ is a potent platelet inhibitor. An increase in TXA_2_ results in the adhesion, aggregation and release of platelets. However, PGI_2_ inhibits platelet aggregation [15], [16]. In our study, aspirin significantly decreased both TXB_2_ and 6-keto-PGF_1α_ levels due to its non-selective and irreversible COX inhibition. Aspirin exerts its antiplatelet activity through inhibition of both COX-1 and COX-2, therefore inhibits not only the production of TXA_2_ in platelets, but also the production of anti-aggregatory PGI_2_ in vessel walls. This phenomenon is referred to as the “aspirin dilemma”[17] and is considered to be a reason underlying the insufficient efficacy and unclear dose–response effects of aspirin [18], [19]. Compared with aspirin, danshensu with higher selectivity for COX-2 is able to normalize the TXA_2_/PGI_2_ balance better, by upregulating 6-keto-PGF_1α_ and downregulating TXB_2_ simultaneously.

Finally, we evaluated the protective effects of danshensu on gastric mucosa. We compared the ulcerogenic effects of danshensu and aspirin on gastric mucosa and demonstrated that danshensu did not cause any apparent ulceration of the gastric mucosa in rats, even at a dose of 60 mg/kg. In contrast, aspirin had a potent ulcerogenic effect at the antithrombotic dose(20 mg/kg) and produced severe hemorrhagic necrotic lesions in the gastric mucosa even at a dose of 200 mg/kg Aspirin-induced gastric lesion was completely inhibited by the coadministration of danshensu. The observed differences in gastric ulcerogenic properties between danshensu and aspirin may be related with the difference in COX-1 selectivity. COX-1-derived PGs are thought to play a dominant role in gastric mucosal defense and cytoprotection and it has been confirmed that selective inhibition of COX-1 alone may not cause ulcers, but inhibition of both COX-1 and COX-2 is required for the development of gastric lesions [20], [21]. Thus, danshensu by selectively inhibit COX-2 only, is considered to be potentially less damaging to the gastrointestinal tract than aspirin that also block COX-1.

In conclusion, our findings suggest that danshensu has potent antithrombotic effects and antiplatelet aggregation activity without inducing GI adverse events. This might be attributed to danshensu's mechanism of its functions as a highly selective COX-2 inhibitor with an ability to normalize TXA_2_/PGI_2_ balance superior to aspirin. Thus, Danshensu may have good prospects in antithrombotic and antiplatelet therapy.

## Materials and Methods

### Chemicals and reagents

Danshensu (purity 98%) was provided by Nanjing Zelang Medical Biological Technology Co. Ltd (Nanjing, China). Aspirin (purity 98%) was produced by Anhui Fengyuan Pharmaceutical Co. Ltd (Anhui, China). Arachidonic acid (AA) was purchased from Sigma-Aldrich Chemical Co. (USA). Heparin sodium was purchased from Jiangsu Wanbang Biochemical Medicine Co. Ltd. (Jiangsu, China). Adrenaline hydrochloride was purchased from Tianjin Jinyao Amino Acids Co. Ltd. (Tianjin, China). COX inhibitor screening assay kits were purchased from Cayman Chemical Company (Ann Arbor, MI, USA). Primary antibodies against COX-1, COX-2, β-actin and horseradish peroxidase (HRP)-conjugated secondary antibody were purchased from Santa Cruz Biotechnology, Inc. The thromboxane B_2_ (TXB_2_) and 6-keto prostaglandin F_1α_ (6-keto-PGF_1α_) radioimmunoassay kits were produced by Tianjin Jiuding Engineering of Medicine and Biology Co. Ltd. (Tianjin, China).

### Animals and treatments

Male Sprague-Dawley rats (weight approximately 300 g, 3–4 months each) were purchased from Shandong Lvye Pharmaceutical Co. Ltd., China (certificate No. SCXK (Lu) 20030008). Male ICR mice (weight approximately 25 g, 8–9 weeks each) were purchased from the Animal Department of the College of Medicine, Beijing University (certificate no. SCXK (Jing) 2006–0008).

Animals were acclimated for at least 1 week to a temperature of 24±1°C and humidity of 55±5%. The animals were maintained with free access to standard diet and tap water. The experimental procedures were approved by the Office of Experimental Animal Management.

Committee of Shandong Province, China (certificate No. SYXK (Lu) 20090015). Rats were given 15, 30 and 60 mg/kg danshensu and 20 mg/kg aspirin intragastrically (i.g.). All drugs were dissolved in 0.9% normal saline as vehicle. The control rats were given 0.9% normal saline. All drugs and saline were administered orally to rats once daily at 9 AM for 7 days. All *in vivo* experiments were conducted 60 min after the treatment.

### Assay of *ex vivo* Platelet Aggregation in Rats

Blood was collected after the last administration of danshensu and saline. All rats were anesthetized with 10% chloral hydrate and blood was collected from the abdominal aorta and anticoagulated with citrate (3.8%; 1 vol anticoagulant: 9 vol blood). Platelet-rich plasma (PRP) was prepared by centrifuging the blood at 1000 rpm for 8 min and at 3000 rpm for 15 min to prepare platelet-poor plasma (PPP). The platelet concentration was adjusted to 1.8−2×10^9^/mL with PPP. Then, 0.3 mL of PRP was placed in a cuvette and stirred with a rotor at 37°C for 5 min, after which 6 µM ADP and 100 µM AA was added. Aggregation was measured with a platelet aggregometer (LBY-NJ4, Pulisheng Instrument Co. Ltd. China). Results were recorded as light transmission at maximal aggregation after the addition of an aggregating agent. Data are expressed as percentage of maximal aggregation.

### *In vivo* arteriovenous shunt thrombosis

Rat arterial-venous shunts (silk thread model) were prepared with 2 2-cm-long polyethylene tubes (1 mm i.d.), linked by a central section (8 cm long; 2 mm i.d.) that contained a 5-cm piece of silk thread and was filled with saline solution that contained heparin 50 U/kg. Rats were anesthetized with chloral hydrate (350 mg/kg, i.p.) and an arterial-venous shunt was placed between the right carotid artery and left jugular vein [22], [23]. After blood was circulated through the shunt for 15 minutes, both ends of the tube were pinched, the silk thread was removed from the shunt tube and the wet and dry weights were measured by subtracting the pre-experiment weight of the 5-cm silk thread.The rate of inhibition of thrombosis formation was calculated as inhibition (%) = (A-A_1_)/A×100%, where A is the thrombus weight of the thrombosis control group and A_1_ is the weight after treatment with the agents. The sham group did not undergo the surgery. All rats fasted overnight before the operation.

### Venous thrombosis model

Venous thrombus formation was induced by inferior vena cava (IVC) ligation to produce a stasis thrombus as described [24], [25]. Rats were anesthetized with chloral hydrate (350 mg/kg, i.p.). After the rats were fixed on a temperature-controlled heating pad (38°C) to maintain body temperature, the abdomen was opened surgically. The intestines were moved gently to one side and covered with saline-moistened gauze and the vena cava was exposed by blunt dissection. One millimeter above the bifurcation of the vena iliaca and vena cava, the free vein was ligated by tightening the proximal and distal segments using 4-0 suture (Shinva Medical, China) to induce blood stasis. The abdominal cavity was closed provisionally and blood stasis was maintained for 4 h. After the abdomen was reopened, the ligated venous segment was excised and opened longitudinally to remove the thrombus. The vein wall and thrombus were divided by blunt or sharp dissection. The isolated thrombus was blotted of excess blood and weighed immediately. Harvested vein samples were homogenized for subsequent biochemical analysis. Sham operation was performed in the control group.

### Measurement of plasma TXB_2_ and 6-keto-PGF_1α_

Blood was drawn from the abdominal aorta at the end of the arteriovenous shunt test period or venous thrombosis model. Plasma was prepared by centrifuging blood at 4000 rpm for 5 min. TXA_2_ and PGI_2_ levels were estimated by measuring their stable hydrolysis products—TXB_2_ and 6-keto-PGF_1α_, respectively—per standard procedures using EIA kits (Cayman Chemical) and expressed as pg/mL.

### Effects of Danshensu on COX activity and expression

The inhibition of COX-1 and COX-2 by danshensu and aspirin was determined using COX inhibitor screening assay kits per the manufacturer's instructions. Danshensu and aspirin (with the same volume of water serving as control) were incubated directly with COX-1 or COX-2 in reaction buffer for 10 min. Then, AA was added as substrate and the reaction continued for another 2 min. Then, 0.1 N HCl and saturated stannous fluoride solution were added immediately to stop the enzymatic reaction. The amount of prostaglandin E_2_ (PGE_2_) that was generated by COX was measured using Cayman ELISA kits. The effects of danshensu and aspirin on COX activity were evaluated by comparing the amounts of PGE_2_ production between reactions with and without the drugs. Inhibitory rates were calculated, based on PGE_2_ production, as follows: Inhibitory rate (%)  =  (PGE_2_ in Control groups-PGE_2_ in drug-treated groups) ×100/PGE_2_ in Control groups.

To measure COX-1 and COX-2 expression, venous walls from the venous thrombosis were analyzed by western blot. Venous samples were homogenized mechanically on ice and centrifuged at 4500 g for 10 minutes at 4°C; total proteins were then collected from the supernatants and the protein content was measured using a BCA protein assay kit.

Samples were separated by SDS-PAGE for immunoblot analysis. After the electrophoresis running for 60 min, proteins were transferred onto polyvinylidenedifluoride (PVDF) membranes. The membranes were blocked with 3% skim milk and saturated in Tris-buffered saline with 1% Tween 20 for 1 h at room temperature. Then, the membrane was incubated with primary anti-COX-1 and anti-COX-2 (1∶300; Cayman Chemical) overnight at 4°C. The membranes were washed 3 times for 5 min each and incubated in horseradish peroxidase (HRP)-conjugated secondary antibody solution for 1 h at room temperature. The blots were washed again and visualized by using an enhanced chemiluminescence (ECL) detection kit (Beyotime Institute of Biotechnology) and exposure to photographic film. Images were collected and the respective bands were quantitated by densitometric analysis using the DigDoc100 program.

### Effects of danshensu on blood stasis

The blood stasis model was established per previous reports [26]. The rats were divided randomly into 6 groups of 10 rats each: normal control, model group given 0.9% normal saline, aspirin (20 mg/kg) and Danshensu (15, 30 and 60 mg/kg). All drugs and saline were administered orally to the rats once per day at 9 AM for 7 days. After the sixth administration, blood stasis was induced by placing the rats in ice-cold water between 2 injections of adrenaline hydrochloride (Adr). All other rats were injected subcutaneously with Adr (0.8 mg/kg), except the control rats, which were injected with 0.9% (w/v) NaCl saline solution. Two hours after the first injection, all rats except the control group were soaked in ice water (0–2°C) for 5 min and reinjected with Adr (0.8 mg/kg) subcutaneously 2 h later to effect blood stasis. Afterward, the rats were placed into cages and fed freely.

Blood samples were collected and anticoagulated with heparin 30 min after the last administration of drugs or saline on the following day. Hemorheologic parameters, including WBV at various shear rates, PV and Hct, were measured by routine laboratory assays. Another aliquot of blood was collected into plastic tubes that contained 3.8% citrate and prepared PRP and PPP as described in *Assay of ex Vivo Platelet Aggregation in Rats.* Platelet aggregation was measured by arachidonic acid-induced.

### Ulcerogenic Effects of Danshensu on Gastric Mucosa

Rats were sacrificed by cervical dislocation and the stomachs were removed immediately to determine the gastric ulcer index after the platelet aggregation assayas described with modifications [27], [28]. The stomach tissues were split longitudinally and opened along the greater curvature. The total length (mm) of visible mucosal lesions in each stomach sample was measured and used to establish an ulcer index. An independent observer scored the macroscopic appearance of the gastric mucosa. The ulcer area was calculated as π/4×a×b mm^2^, where a is the long axis and b is the short axis. The ulcer area was expressed as an ulcer index.

### Effects of danshensu on aspirin-induced gastric lesion in mice

To investigate the effect of danshensu on aspirin-induced gastric lesions, male ICR mice were randomly divided into 4 groups. Group 1 received 1% carboxymethylcellulose in water as vehicle, Group 2 received danshensu treatment (120 mg/kg body weight), Group 3 received aspirin treatment (200 mg/kg body weight, suspended in 1% carboxymethylcellulose in water) and Group 4 received treatment of aspirin and danshensu. All drugs were administered orally once per day at 9 AM for 7 days. The mice were anesthetized with ether and sacrificed. One hour after the last administration, the isolated stomach was ligated at pylorus and cardia ends, filled with 1.5 mL of 2% formalin and immersed in 2% formalin for 15 min. Then, the stomach was cut along the greater curvature and the lengths of lesions on the stomach wall were measured as described [29].

### Statistical analysis

Data were analyzed using SigmaStat 3.1 (SPSS Inc.; Chicago, IL) and expressed as mean±standerd error of means (SEM). P<0.05 was considered to be statistically significant.
